# Editorial: Enabling Wearable Brain Technologies - Methods and Applications

**DOI:** 10.3389/fnhum.2021.722388

**Published:** 2021-09-10

**Authors:** Marcin Wozniak, Victor Hugo C. de Albuquerque, Adel Said Elmaghraby

**Affiliations:** ^1^Faculty of Applied Mathematics, Silesian University of Technology, Gliwice, Poland; ^2^Graduate Program on Teleinformatics Engineering, Federal University of Ceará, Fortaleza, Brazil; ^3^Computer Science and Engineering, JB Speed School of Engineering, University of Louisville, Louisville, KY, United States

**Keywords:** computational intelligence, computational intelligence method, brain understanding and analysis, augmented reality and human machine interaction, augmented reality, deep learning, deep learning-artificial neural network, internet of wearable brain things

## Introduction

Modern technological advances in portable technologies have made it possible to study the brain in its entirety, to promote convalescence after an accident and to help in the treatment of various brain diseases and disorders, to develop a greater perception of the world around us, etc. All of this is possible because the results of modern technologies allow us to better understand how the brain works. Readings and images from different sensors can be processed as digital values (Zhu et al.; Zheng et al.; Zhang et al.), dedicated image forms or even sophisticated waves using advanced methodologies (Wang et al.; Zhang et al.). A variety of new aspects is studied in modern sciences to better understand how the brain works.

This Research Topic aims to find answers to the following questions: Is it possible to use brain to control some devices? How the processes of control and interaction through modern technology reflect thinking and functions of the brain? Do we see any clues for potential disorder from readings of brain sensors? Do we have yet good methods to analyze brain signals and use them for prediction? Is modern science able to simulate brain and its functions?

This Research Topic presents a collection of research papers covering an open cross-field junction between technology, methodology, science and didactics to enable professional discussion and presentation of innovative and efficient ideas to maximize any possible benefits of the research to the society, at a technological and methodological level.

## Research Topic Coverage

This Research Topic received a wide acknowledgments among research communities in various parts of the World. The authors of accepted publications presented articles covering latest advances on Internet of Things, deep learning for neuroimage and neurosignal processing, Virtual and Augmented Reality for neuroscience, multimodal data fusion, and various interesting real world applications and simulation.

In Wang et al. was presented a research on the model of Electroencephalography (EEG) signal from electrodes placed on the scalp. Experiment has shown how to approach potential disorders of the auditory function within the brain by using proposed Random Stimulation Rate (RSR) method which integrates a random interval between two adjacent stimuli. As a result an early diagnoses of auditory pathway abnormities was proposed.

In Flynn et al. was presented a model of automatic emotion recognition by using neural network approach. In the research Authors analyzed combination of methodology underpinned by psychology and latest technology like iMotions biometric research platform to formulate model of relations between automatically and interactively captured responses of participants in a form of International Affective Picture System (IAPS) standard.

In Zhu et al. was proposed Hierarchical Attentive Decoding (HAD) model where by exploring knowledge associations in sequence-to-sequence classification framework Authors proposed a model of adaptive assignment of multiple species from input texts.

In Zhang et al. was presented a new idea for automatic diagnosis of patients with alcoholism by analyzing neural activity from electroencephalogram (EEG) signals in a two-dimensional perspective. Model works in a form of Transfer Learning with Convolutional Neural Network (CNN). Authors tested various configurations to find the best architecture.

In Zheng et al. was presented a model to estimate mental fatigue by proposed Steady-State Visual Evoked Potential (SSVEP) from six stimulus paradigms: reverse vertical sinusoidal gratings, reverse horizontal sinusoidal gratings, reverse vertical square-wave gratings, brief-onset vertical sinusoidal gratings, reversal checkerboards, and oscillating expansion–contraction concentric rings.

In Ptito et al. was presented a discussion on new trends and possibilities sourced in research on technology and neuroscience to help blind people by using sensory substitution or cross-modal plasticity for brain to support vision sense.

In Zhang et al. was presented a novel non-contact sensing technique to detect sensory ataxia and cerebellar ataxia, which reveal in poor body coordination and balance disorder. Authors present Romberg's test and gait analysis model which collect information for machine learning based classifiers. Results have shown high potential for further research and development.

All presented articles show important ideas for solving medical problems in brain sensing and activity by applied models of Artificial Intelligence. Editors are happy to present this collection to the scientific community, and hope that it will start a new trend on neuroscience development.

## Author Contributions

All authors listed have made a substantial, direct and intellectual contribution to the work, and approved it for publication.

## Conflict of Interest

The authors declare that the research was conducted in the absence of any commercial or financial relationships that could be construed as a potential conflict of interest.

## Publisher's Note

All claims expressed in this article are solely those of the authors and do not necessarily represent those of their affiliated organizations, or those of the publisher, the editors and the reviewers. Any product that may be evaluated in this article, or claim that may be made by its manufacturer, is not guaranteed or endorsed by the publisher.

